# Efficacy of mesenchymal stem cell therapy for sepsis: a meta-analysis of preclinical studies

**DOI:** 10.1186/s13287-020-01730-7

**Published:** 2020-06-03

**Authors:** Xue-Yi Sun, Xian-Fei Ding, Huo-Yan Liang, Xiao-Juan Zhang, Shao-Hua Liu, Bing-Han, Xiao-Guang Duan, Tong-Wen Sun

**Affiliations:** General ICU, Henan Key Laboratory of Critical Care Medicine, The First Affiliated Hospital of Zhengzhou University, Zhengzhou Key Laboratory of Sepsis, Zhengzhou, 450052 China

**Keywords:** Mesenchymal stem cell therapy, Sepsis, Meta-analysis, Preclinical studies

## Abstract

**Background:**

Multiple studies have reported that mesenchymal stem cell (MSC) therapy has beneficial effects in experimental models of sepsis. However, this finding remains inconclusive. This study was performed to systematically determine the connection between MSC therapy and mortality in sepsis animal models by pooling and analyzing data from newly published studies.

**Methods:**

A detailed search of related studies from 2009 to 2019 was conducted in four databases, including MEDLINE, EMBASE, Cochrane Library, and Web of Science. After browsing and filtering out articles that met the inclusion criteria for statistical analysis, the inverse variance method of the fixed effects model was used to calculate the pooled odds ratios (ORs) and their 95% confidence intervals (CIs).

**Results:**

Twenty-nine animal studies, including 1266 animals, were identified. None of the studies was judged to have a low risk of bias. The meta-analysis demonstrated that MSC therapy was related to a significantly lower mortality rate (OR 0.29, 95% CI 0.22–0.38, *P* < 0.001). Subgroup analyses performed based on the MSC injection dose (< 1.0 × 10^6^ cells, OR = 0.33, 95% CI 0.20–0.56, *P* < 0.001; 1.0 × 10^6^ cells, OR = 0.24, 95% CI 0.16–0.35, *P* < 0.001) and injection time (< 1 h, OR = 0.24, 95% CI 0.13–0.45, *P* < 0.001; 1 h, OR = 0.28, 95% CI 0.17–0.46, *P* < 0.001) demonstrated that treatment with MSCs significantly reduced the mortality rate of animals with sepsis.

**Conclusion:**

This up-to-date meta-analysis showed a connection between MSC therapy and lower mortality in sepsis animal models, supporting the potential therapeutic effect of MSC treatment in future clinical trials. The results in this study contradict a previous meta-analysis with regards to the ideal dose of MSC therapy. Thus, further research is required to support these findings.

## Background

Sepsis is a life-threatening organ dysfunction caused by a host of uncontrolled responses to infection [1]. Sepsis is a common cause of patient hospital admission and death in the intensive care unit (ICU) [2], causing one third to one half of all deaths in hospital [3] and killing more than six million individuals worldwide each year [4]. Currently, there is no effective treatment for sepsis, and its management mainly focuses on controlling the source, as well as antibiotic application along with organ function support [5]. Due to the high mortality of sepsis, there is an unmet need for identifying considerable medical therapy for sepsis.

   Mesenchymal stem cell (MSC) therapy has recently gained more attention due to the easy and fast isolation and expansion of MSCs in comparison to other stem cells, such as embryonic stem cells [6]. MSCs have multi-directional differentiation potential and can differentiate into many types of cells, such as adipocytes, chondroblasts, osteoblasts, and tissue macrophage-like cells, making MSCs one of the most necessary and promising sources of new clinical treatment [7, 8]. Related studies have been widely performed in the context of many different diseases, such as graft versus host disease, progressive multiple sclerosis, diabetes, stroke, bronchopulmonary dysplasia, cardiomyopathy, and osteoarthritis [9]. Undoubtedly, because these cells have immunomodulatory, anti-inflammatory, antibacterial, and differentiation properties, MSCs are currently one of the most promising treatment options [9]. Despite the multiple studies conducted over the past decade, further research is still needed to confirm whether MSCs have definite beneficial effects on the management of sepsis.

    Several animal studies that focus on sepsis have been reported [10–33], but these studies use different experimental designs and yield contradictory results. Thus, further preclinical studies are still required to evaluate the risks of new treatments and predict the safety or effectiveness of the therapy. In addition, such research can provide references and recommendations for unresolved issues in clinical stem cell therapy. Therefore, we conducted a systematic review of the literature and meta-analysis to assess the effectiveness of MSC treatment in animals with sepsis.

## Methods

This meta-analysis was performed according to the Preferred Reporting Items for Systematic Reviews and Meta-Analyses (PRISMA) criteria [34]. The analysis of data available in published articles does not require ethical approval and patient consent. All supporting data is provided in this article and supplemented online.

### Data sources and search strategies

The researchers conducted a systematic literature search using four databases, including MEDLINE, EMBASE, Cochrane Library, and Web of Science, to screen for targeted studies on the efficacy of MSCs in treating sepsis. The detailed search strategy is shown in Additional file 1: Table S1. The last search was updated on October 31, 2019. English was chosen as our search language. Later, all lists of references from the related articles (reviews, systematic reviews, meta-analyses, and included studies) were scanned by hand to retrieve additional studies that were not listed in the above databases. Two independent investigators blindly performed the literature search (XY Sun and XF Ding).

### Eligibility criteria

The studies included in this meta-analysis fulfilled the following criteria: (1) the study evaluated the efficacy of MSC treatment in sepsis animal models (all species and sexes), (2) the study was written in English, (3) The study involved animal models of sepsis or endotoxemia, and (4) the study reported the evaluation index, including mortality. If more than one article contained overlapping data, the most informative or recent article was used.

   The exclusion criteria were as follows: (1) the MSCs used in the study were differentiated, altered, or designed to overexpress or express specific genes; (2) the animal models suffered from sepsis but did not accept MSC therapy; (3) the animal models had other comorbidities; (4) the animals did not suffer from sepsis; (5) the study had insufficient data to obtain endpoint outcomes of interest; (6) the study did not have a control group; and (7) the study was duplicated.

### Study selection and data extraction

Based on the search strategy to identify studies that met the above inclusion criteria, XY Sun and SH Liu retrieved the title and/or abstract of the studies from the database search, as well as from the supplementary resources. The main data disclosure was carried out separately by XJ Zhang and TW Sun. Any disagreements were resolved through discussions with B Han and XG Duan. The collected data was as follows: author and publishing year, animal characteristics (species, gender, sample size, and model), intervention characteristics (origin, dose, route, and timing of the MSC treatment), follow-up (time to observe results after MSC administration), and our primary measures related to secondary outcomes. If available, the odds ratio (OR) and its related 95% confidence interval (CI) were extracted directly from the original article. Otherwise, the OR and 95% CI were calculated from the individual patient data in the study.

### Assessment of risk of bias (RoB)

The RoB of the experimental animal studies was evaluated using the Systematic Review Centre for Laboratory Animal Experimentation (SYRCLE) RoB tool [35]. This tool is based on the Cochrane RoB tool and has been adjusted for aspects of bias that play a specific role in animal intervention studies. Widespread adoption and implementation of this tool will facilitate and improve the critical appraisal of evidence from animal studies. This may subsequently enhance the efficiency of translating animal research into clinical practice and increase awareness of the necessity for improving the methodological quality of animal studies. The resulting tool for animal studies contains 10 entries. These entries are related to six types of bias. Entries in this tool are as follows: selection bias (sequence generation, baseline characteristics, and allocation concealment), performance bias (random accommodation and blinding), detection bias (random outcome assessment and blinding), attrition bias (incomplete outcome data), reporting bias (selective outcome reporting), and other sources of bias. For each included study, the RoB was scored as high, low, or unclear.

### Primary outcomes

The main study outcome of this meta-analysis was mortality.

### Statistical analysis

The sepsis mortality rate was the OR taken as the effect size, and each effect size was expressed with a 95% CI. In addition, *χ*^2^ and *I*^2^ tests were used to measure heterogeneity, where a *P* > 0.1 and *I*^2^ < 50% was considered to be low heterogeneity [36, 37]. If there was no heterogeneity, the inverse variance method of the fixed effects model was used for meta-analysis [38, 39]. If heterogeneity was present, subgroup analysis was performed to explore the potential sources of the heterogeneity and to consider whether meta-analysis could be conducted using a random effects model. Begg’s funnel plot and Egger’s linear regression were used to assessing potential publication bias [40]. All analyses were performed using Stata 14.0 statistical software (Stata Corp LP, College Station, Texas 77,845, USA). Differences with a *P* < 0.05 (two-sided) were considered statistically significant.

## Results

### Study selection

**Fig. 1:**
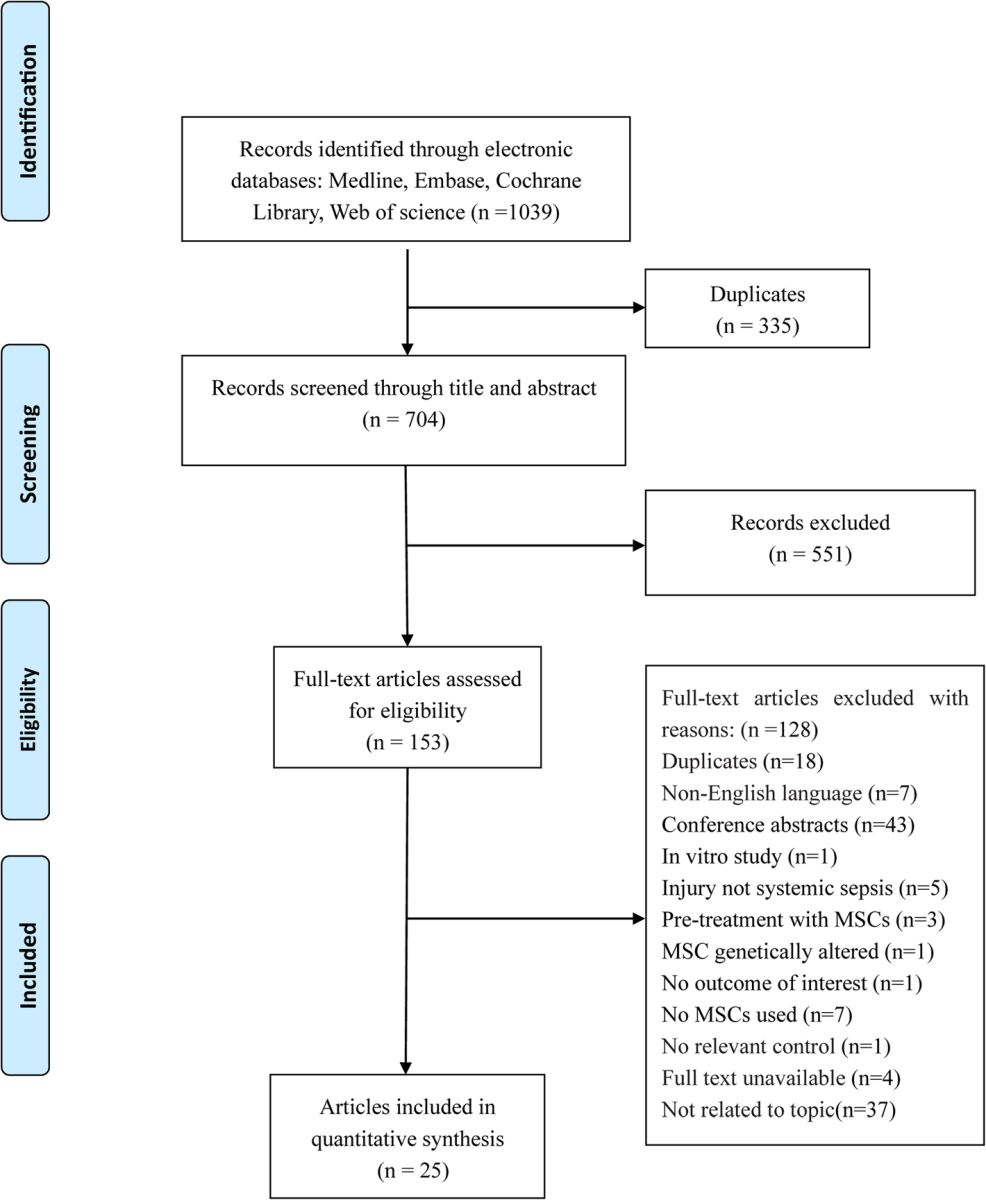
Flow diagram of the study selection

According to the search strategy, a total of 1039 studies were identified and 704 studies remained after deleting duplicates. After preliminarily screening the titles and abstracts, 128 articles reporting the potential of MSCs for the treatment of sepsis were isolated for full-text review. Ultimately, 25 articles [10–33, 41] involving 1266 animals were included in this meta-analysis. The inclusion process and the reasons for the removal of certain studies are shown in Fig. 1.

### Study characteristics

The basic characteristics of the included studies are shown in Table 1. The articles were published between 2009 and 2019, with sample sizes ranging from 14 to 139. The sepsis animal models in most studies were induced by cecal ligation and puncture (CLP) or intraperitoneal injection of lipopolysaccharide (LPS). The animals used included rats and mice. Regarding the characteristics of the MSCs used for intervention, the MSCs were mainly derived from human or rat bone marrow, adipose-derived mesenchymal tissue, or human umbilical cord blood-derived mesenchymal tissue, with intervention doses ranging from 10^5^ to 10^7^ MSCs. Most of the MSCs were injected intravenously or intraperitoneally within a few hours of the induction of the sepsis animal models. In addition, four of the articles [10, 13, 29, 32] included multiple studies. Thus, the meta-analysis included a total of 29 animal studies with 1266 animals.

**Table 1:** General characteristics of preclinical studies investigating the efficacy of mesenchymal stem cells in models of sepsis

Author year Country	Species, Strain, Gender	No. of treated animals	No. of controls	Sepsis model	MSC source, Compatibility	MSC Dose	Time of delivery post-sepsis induction	MSC route	Control
Gonzalez-Rey et al. (2009)A Spain [10]	Mouse BALB/c, NR	18	10	CLP	Adipose, Xenogenic or Allogeneic	1.0×10^6^	4 hours	IP	DMEM
Gonzalez-Rey et al. (2009)B Spain	Mouse BALB/c, NR	20	10	LPS (i.p.)	Adipose, Xenogenic or Allogeneic	1.0 × 10^6^ or 3.0 × 10^5^	0.5 hours	IP	DMEM
Nemeth et al. (2009) United States [11]	Mouse C57BL/6, M	90	45	CLP	Bone marrow, Allogeneic	1.0×10^6^	0 or 1 hour	IV	PBS
Bi et al. (2010) China [12]	Mouse C57BL/6, NR	10	10	CLP	Bone marrow, Xenogenic	1.0×10^6^	1 1 hours	IV	PBS
Mei et al. (2010)A Canada [13]	Mouse C57BL/6J, F	29	29	CLP	Bone marrow, Syngeneic	2.5×10^5^	6 hours	IV	NS
Mei et al. (2010)B Canada	Mouse C57BL/6J, F	15	20	CLP	Bone marrow, Syngeneic	2.5×10^5^	6 hours	IV	NS
Liang et al. (2011) China [14]	Rat Wistar, F	15	15	LPS (i.v.)	Bone marrow, Syngeneic	1.0×10^6^	2 hours	IV	NS
Chang et al. (2012) China [15]	Rat SPD, M	16	16	CLP	Adipose, Autologous	3× 1.2×10^6^	0.5, 6 then 18	IP	NS
Krasnodembskaya et al. (2012) USA [16]	Mouse C57BL/6J, M	34	69	P. aeruginosa (i.p.)	Bone marrow, Xenogenic	1.0×10^6^	1 hour	IV	PBS
Li et al. (2012) China [17]	Rat SPD, M	20	40	LPS (i.p.)	Umbilical cord, Xenogenic	5.0×10^5^	1 hour	IV	NS
Hall et al. (2013) USA [18]	Mouse BALB/c, M	26	35	CLP	Bone marrow, Syngeneic	1× 5.0 ×10^5^ + 2 ×2.5× 10^5^	2 then 24 then 48 hours	IV	PBS
Zhao et al. (2013) China [19]	Rat SPD, F	24	27	LPS (i.v.)	Bone marrow, Syngeneic	2.5×10^6^	2 hours	IV	NS
Chao et al. (2014) Taiwan [20]	Rat Wistar, M	20	10	CLP	Bone Marrow or Umbilical Cord, Xenogenic	5.0×10^6^	4 hours	IV	PBS
Kim et al. (2014) Canada [21]	Mouse C57BL/6, M	73	66	SEB+ (i.p)	Bone marrow, Syngeneic	2.5×10^5^	3 hours	IV	PBS
Luo et al. (2014) China [22]	Mouse C57Bl/6, M	20	20	CLP	Bone marrow, Syngeneic	1.0×10^6^	3 hours	IV	NS
Pedrazza et al. (2014) Brazil [23]	Mouse C57BL/6, M	15	15	E. coli (i.p.)	Adipose, Syngeneic	1.0×10^6^	0	IV	PBS
Sepulveda et al. (2014) Spain [24]	Mouse BALB/c, M	30	10	LPS (i.p.)	Bone Marrow, Xenogenic	1.0×10^6^	0.5 hour	IP	PBS
Zhao et al. (2014) China [25]	Mouse C57BL/6, M	12	12	CLP	Umbilical cord, Xenogenic	1.0×10^6^	1 hour	IV	NS
Zhou et al. (2014) China [26]	Mouse NOD SCID, M	7	7	LPS+ (i.p.)	Umbilical Cord, Xenogenic	2.0×10^6^	6 hours	IV	No treatment
Yang et al. (2015) China [28]	Mouse NOD SCID, M	10	10	LPS+ (i.p.)	Umbilical cord, Xenogenic	5.0×10^5^	0	IV	DMEM
Francisca et al.(2015) Chile [27]	Mouse C57BL6/j, NR	21	16	CLP	menstrual fluid, Xenogenic	7.5 × 10^5^	3 hours	IV/IP	NS
Hao Ou et al.(2015)A China [29]	Mouse SPF BALB/c, NR	7	14	LPS	Adipose, Allogeneic	1.0 × 10^7^	5 mins	IV	NS
Hao Ou et al.(2015)B China	Mouse SPF BALB/c, NR	9	14	LPS	Bone marrow, Allogeneic	1.0 × 10^7^	5 mins	IV	NS
Pei-Hsun Sung et al.(2017) Taiwan, China [30]	Rats SPD, M	16	16	CLP	Adipose, Autologous	1.2×10^6^	3 hours	IV	NS
Xujing Liang et al.(2019) China [33]	Rats SPD, M	10	10	CLP	Umbilical cord, Xenogenic	2.5×10^6^	0	IV	PBS
Xian-Fei Ding et al.(2019) China [31]	Rats SPD, M	30	30	CLP	Adipose, Allogeneic	1.0×10^6^	1 hour	IP	NS
Mirjana Jerkic et al.(2019)A Canada [32]	Rats SPD, M	12	15	E. coli	Umbilical cord, Xenogenic	1.0× 10^7^	1 hour	IV	PBS
Mirjana Jerkic et al.(2019)B Canada	Rats SPD, M	11	15	E. coli	IL-10 UC-MSCs, Xenogenic	1.0 × 10^7^	1 hour	IV	PBS
Huoyan Liang et al.(2019) China [41]	Rats SPD, M	20	20	CLP	Adipose, Allogeneic	1.0×10^6^	1 hour	IP	NS

*CLP* Cecal ligation and puncture, *DMEM* Dulbecco’s modified Eagle’s medium, *i.p.* Intraperitoneal, *i.v.* Intravenous, *LPS* Lipopolysaccharide, *NR* Not reported, *NS* Normal saline, *PBS* Phosphate buffered saline, *SEB* Staphylococcal enterotoxin B, *SPD* Sprague Dawley, *M* Male, *F* Female, *E. coli* Escherichia coli, *P. aeruginosa* Pseudomonas aeruginosa

### Assessment of RoB

Table 2 shows the RoB assessment results for the included studies. No study was considered to have low RoB. The included studies showed similarities between the baselines of the experimental and control groups, reducing the risk of selection bias in accordance with the animal characteristics. Despite the random allocation of experimental and control subjects, none of the studies clearly described the generation of random sequences. Therefore, the RoB was judged to be “unclear” in the sequence generation domain of all the included studies. However, no study properly described the method of concealed allocation, animals were randomly fed, researchers were blind to the interventions that each animal received, a randomized outcome evaluation was reported, and, in one study [21], the blindness of the evaluator was recorded. Using the provided signal questions, the risk of attrition bias and reporting bias in all of the included studies was low. The data in two of the studies [12, 26] was inadequate. Three of the studies [10, 13, 15] may have had other problems that pose a high RoB, including pollution, experimental design, and so on. In addition, we did not identify any additional sources of bias and bias tools for systematic risks that were not covered.

**Table 2:** SYRCLE Risk of Bias Assessment for included studies

Author (Year)	Random sequence generation?	Groups similar at baseline?	Allocation concealed?	Animals randomly housed?	Blinding of caregivers and/or examiners?	Random selection for outcome assessment?	Blinding of outcome assessor?	Incomplete outcome data addressed?	Free from selective outcome reporting?	Free from other bias?
Gonzalez-Rey et al.(2009) [10]	U	U	U	U	U	U	U	L	L	H
Nemeth et al. (2009) [11]	U	U	U	U	U	U	U	L	L	L
Bi et al. (2010) [12]	U	U	U	U	U	U	U	H	L	L
Mei et al. (2010) [13]	U	U	U	U	U	U	U	L	L	H
Liang et al. (2011) [14]	U	U	U	U	U	U	U	U	L	L
Chang et al. (2012) [15]	U	U	U	U	U	U	U	U	L	H
Krasnodembskaya et al.(2012) [16]	U	U	U	U	U	U	U	U	L	L
Li et al. (2012) [17]	U	U	U	U	U	U	U	U	L	L
Hall et al. (2013) [18]	U	U	U	U	U	U	U	U	L	L
Zhao et al. (2013) [19]	U	U	U	U	U	U	U	U	L	L
Chao et al. (2014) [20]	U	U	U	U	U	U	U	U	L	L
Kim et al. (2014) [21]	U	U	U	U	U	U	H	U	L	L
Luo et al. (2014) [22]	U	U	U	U	U	U	U	U	L	L
Pedrazza et al. (2014) [23]	U	U	U	U	U	U	U	U	L	L
Sepulveda 2014 [24]	U	U	U	U	U	U	U	U	L	L
Zhao et al. (2014) [25]	U	U	U	U	U	U	U	U	L	L
Zhou et al. (2014) [26]	U	U	U	U	U	U	U	H	L	L
Yang et al. (2015) [28]	U	U	U	U	U	U	U	U	L	L
Francisca et al. (2015) [27]	U	U	U	U	U	U	U	L	L	L
Hao Ou et al. (2015) [29]	U	U	U	U	U	U	U	L	L	L
Pei-Hsun Sung et al. (2017) [30]	U	U	U	U	U	U	U	L	L	L
Xujing Liang et al. (2019) [33]	U	U	U	U	U	U	U	L	L	L
Xian-Fei Ding et al. (2019) [31]	U	U	U	U	U	U	U	L	L	L
Mirjana Jerkic et al. (2019) [32]	U	U	U	U	U	U	U	U	U	U
Huoyan Liang et al. (2019) [41]	U	U	U	U	U	U	U	L	L	L

*H* High risk of bias, *L* Low risk of bias, *U* Unclear risk of bias

### Effect of MSC therapy on sepsis

**Fig. 2:**
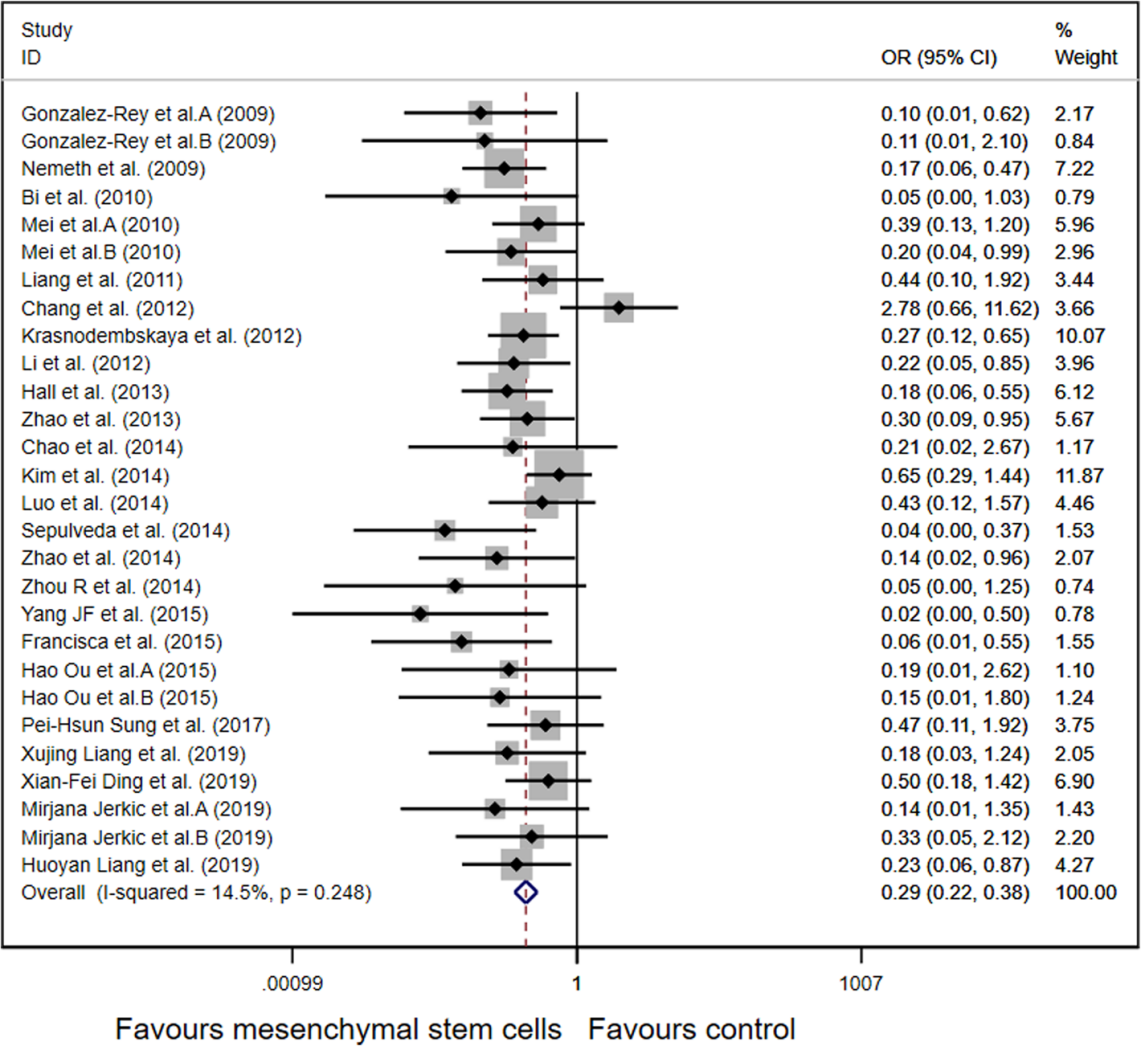
Forest plot summarizing the effects of mesenchymal stem cell therapy on the mortality of preclinical models of sepsis and endotoxemia

A total of 29 animal studies involving 1266 animals were used in this meta-analysis and reported animal mortality rates. Heterogeneity test results showed *I*^2^ = 14.5% and *P* = 0.248, indicating that the heterogeneity between the studies was low; thus, a fixed effects model was used. As shown in Fig. 2, the pooled results demonstrated that the mortality of the animals after MSC treatment was significantly reduced (OR 0.29, 95% CI 0.22–0.38, *P* < 0.001).

### Sensitivity analysis

Sensitivity analysis was performed by eliminating any one of the 29 studies one by one, and assessing whether the combined effect value still fell within the total combined effect value of the 95% CI, indicating that the results of the meta-analysis were stable.

### Subgroup analysis

Subgroup analysis was performed based on the animal species and model, as well as the MSC source, dose, injection time, and injection route. Both injection doses of 1.0 × 10^6^ MSCs (OR = 0.24, 95% CI 0.16–0.35, *P* < 0.001) and less than 1.0 × 10^6^ MSCs (OR = 0.33, 95% CI 0.20–0.56, *P* < 0.001), as well as the injection times of less than 1 h (OR = 0.24, 95% CI 0.13–0.45, *P* < 0.001) and 1 h (OR = 0.28, 95% CI 0.17–0.46, *P* < 0.001) after sepsis induction in the animal models, significantly reduced the mortality rate (Additional files 2 and 3: Figs. S1 and S2). Intravenous injection of MSCs significantly reduced the mortality rate of the sepsis animal models (OR = 0.28, 95% CI 0.21–0.38, *P* < 0.001) compared to intraperitoneal administration (OR = 0.37, 95% CI 0.20–0.69, *P* < 0.001) (Additional file 4: Fig. S3). MSCs administered to mice were more effective (OR = 0.24, 95% CI 0.17–0.34, *P* < 0.001) than MSCs administered to rats (OR = 0.39, 95% CI 0.25–0.60, *P* < 0.001) (Additional file 5: Fig. S4). However, there was no significant difference between the mortality rates of the CLP-induced sepsis animal model (OR = 0.29, 95% CI 0.20–0.42, *P* < 0.001) and the no-CLP sepsis animal model (OR = 0.29, 95% CI 0.22–0.38, *P* < 0.001) (Additional file 6: Fig. S5). Furthermore, umbilical cord-derived MSCs significantly reduced the mortality rate of sepsis in the animal models (OR = 0.14, 95% CI 0.06–0.32, *P* < 0.001). Only one study [27] showed that menstrual fluid-derived MSCs are efficacious for sepsis treatment (OR = 0.06, 95% CI 0.01–0.55, *P* < 0.001); however, this finding should be interpreted cautiously (Additional file 7: Fig. S6). Additionally, MSCs administered to male animal models (OR = 0.31, 95% CI 0.23–0.43, *P* < 0.001) were more beneficial than those administered to female animal models (OR = 0.33, 95% CI 0.17–0.63, *P* < 0.001) (Additional file 8: Fig. S7).

### Publication bias

Begg’s funnel plot was used to test the potential bias of the literature. The results of the Begg’s funnel plot suggested that publication bias was likely present. The Egger’s test also suggested asymmetry in the funnel plot (*P* = 0.002).

## Discussion

Systematic reviews play a vital role in assessing whether preclinical data can be applied to clinical practice. Combining such reviews with meta-analyses enables a more comprehensive and objective assessment of scientific results. Multiple preclinical studies of sepsis animal models revealed that MSCs could improve sepsis and decrease the mortality rate of sepsis. However, thus far, MSC therapy has not been used in the clinical treatment of patients with sepsis. A previous meta-analysis [42] collected and evaluated preclinical evidence regarding the use of MSCs in animal models of sepsis and demonstrated that MSC therapy could reduce the odds of death. The updated meta-analysis presented here includes another seven high-quality studies [27, 29–33, 41] that were mainly published in recent 3 years, and confirms the potential therapeutic efficacy of MSCs for reducing the mortality rate of sepsis in animal models, thus, providing possibilities for MSC therapy in preclinical studies of sepsis. To our knowledge, two small clinical phase 1 trials [43, 44] have been performed to evaluate the safety and feasibility of MSC therapy in sepsis and septic shock patients. These trials showed no serious clinical or physiological safety signals, implying that MSC treatment was tolerated and safe for administration in critical patients with septic shock.

In our study, MSC therapy significantly improved the mortality rate of sepsis animal models, supporting the potential use of MSC therapy in preclinical studies of sepsis. Moreover, our study revealed that umbilical cord-derived MSCs significantly reduced the mortality rate of animals with sepsis; however, there is one study [27] that indicated that menstrual fluid-derived MSCs were also efficacious. However, given the lack of related reports, further research is required to confirm these findings. Numerous subgroup analyses were performed based on the MSC injection dose (< 1.0 × 10^6^ MSCs or 1.0 × 10^6^ MSCs) and injection time (< 1 h or 1 h) and demonstrated that MSCs significantly reduced the mortality rate of animals with sepsis. The effectiveness of intravenous injection of MSCs was greater than that of intraperitoneal administration. Furthermore, MSC administration in mice was more effective than MSC administration in rats. Additionally, the beneficial effects of MSCs in male animal models were greater than in female animal models. The sex difference might be an essential factor for MSC administration. Compared to the previously published meta-analysis [42], we obtained similar results with regards to the ideal source of MSCs, the optimal injection time, and the ideal route of MSC injection. However, concerning the MSC therapy dose, our analysis yielded a different conclusion that the ideal dose is no more than 1.0 × 10^6^ MSCs or equal to 1.0 × 10^6^ MSCs, contradicting the conclusions reported in the previously published meta-analysis [42], where more than or equal to 1.0 × 10^6^ MSCs was indicated as the ideal dose. Thus, further research is required to explore the ideal dose of MSCs for sepsis treatment.

Previous studies show that the early stages of sepsis are characterized by an excessive inflammatory state due to the overproduction of pro-inflammatory mediators, which triggers end-organ dysfunction and damage [45]. MSCs may have the ability to increase anti-inflammatory cytokines and reduce pro-inflammatory cytokines [10, 20, 21, 23]. Liang et al. [41] proved that adipose-derived mesenchymal stem cells (ADMSCs) can reduce liver damage and inflammation through soluble tumor necrosis factor receptor 1 (sTNFR1), and more importantly, ADMSCs can significantly improve the survival rate of rats with CLP-induced sepsis. Two studies [33, 46] suggest that the expression levels of various pro-inflammatory cytokines, such as tumor necrosis factor (TNF)-α and interleukin (IL)-6, are elevated in CLP rats or LPS-treated Kupffer cells. Moreover, these studies found that MSCs have inhibitory effects on the sepsis-induced overexpression of TNF-α and IL-6 and enhancing effects on IL-4 and IL-10 expression in rats with sepsis and LPS-treated Kupffer cells. Contrastingly, a recent study published in 2019 [6] yielded opposite results that suggested that MSCs may not reduce the systemic inflammatory response, but can reduce organ damage. Therefore, further studies should be carried out to explore the potential mechanism of MSC therapy.

The advantages of the meta-analysis conducted here are apparent. Firstly, our study provides an up-to-date meta-analysis of the effectiveness of MSCs in sepsis animal models. Although a previously published meta-analysis [42] assessed the effectiveness of MSCs in sepsis animal models, the meta-analysis presented here includes seven recently published high-quality studies [27, 29–33, 41]. Out of all the included studies, only one study showed no positive effect of MSC therapy on sepsis animal models (OR = 2.78, 95% CI 0.66–11.62, *P* < 0.001). Secondly, we conducted a thorough and careful literature search that obeyed publishing protocols to ensure a strict reviewing procedure. Thirdly, numerous subgroup analyses based on the various animal models, source of MSCs, route of MSC administration, dose of MSCs, and timing of MSC administration were conducted to increase the accuracy of our findings. Finally, the main results of this investigation are generally useful for later preclinical and clinical trials for sepsis treatment.

However, this meta-analysis also has several limitations. The funnel plots and Egger’s linear regression tests revealed that the study might contain publication bias. All included studies were limited to published studies; thus, unpublished data was omitted, possibly reducing the accuracy of our results. Although our best efforts were made to conduct a comprehensive search of the current literature, it is possible that some related studies were missed. In addition, due to the strict inclusion criteria, the meta-analysis was limited to relatively small data sets, and these studies were subject to external publication bias. Finally, it is hard to comment on the clinical safety of MSC treatment. While immunogenicity is unrelated to MSC therapy [43, 44], other significant risks still exist. Previous meta-analyses have shown no direct relationship between MSC administration and acute poisoning, systemic failure, malignancy, or death [47–49]. Regarding the safety and effectiveness of MSCs in the treatment of sepsis, phase 1 clinical trials have been conducted [43, 44], but MSCs have not yet been used for the clinical treatment of sepsis. To our knowledge, a recent large-animal study [50], using the animal models of pigs, demonstrated that MSCs were not capable of reducing the mortality induced by sepsis. Concerning its small sample size, more large-animal studies are needed. In spite of the current limitations, to some extent, our results represent the trends in this research area.

## Conclusion

The present meta-analysis included 25 studies involving a total of 1266 animals and indicated that MSC therapy for sepsis is associated with lower mortality in preclinical studies. In this regard, large-animal studies and large-scale animal studies are needed. Furthermore, large-scale, randomized, prospective clinical trials are required to determine the effectiveness and efficiency of MSCs for the treatment of sepsis in patients, thereby, substantially improving present treatment protocols.

## Supplementary information

**Additional file 1: Table S1.** The detailed search strategy.

**Additional file 2: Fig. S1.** Forest plot summarizing the relationship between mesenchymal stem cell dose and mortality in preclinical models of sepsis and endotoxemia.

**Additional file 3: Fig. S2.** Forest plot summarizing the relationship between mesenchymal cell therapy timing of administration and mortality in preclinical models of sepsis and endotoxemia.

**Additional file 4: Fig. S3.** Forest plot summarizing the relationship between mesenchymal stem cell administration route (intravenous versus intraperitoneal injection) and mortality in preclinical models of sepsis and endotoxemia.

**Additional file 5: Fig. S4.** Forest plot summarizing the relationship between mesenchymal stem cell-treated animal model species (rat versus mouse) and mortality in preclinical models of sepsis and endotoxemia.

**Additional file 6: Fig. S5.** Forest plot summarizing the relationship between preclinical models of sepsis and endotoxemia (i.e. cecal ligation and puncture versus live bacteria or bacterial product administration) and mortality following treatment with mesenchymal stem cells.

**Additional file 7: Fig. S6.** Forest plot summarizing the relationship between mesenchymal stem cell source and mortality in preclinical models of sepsis and endotoxemia.

**Additional file 8: Fig. S7.** Forest plot summarizing the relationship between animal sex and mortality in preclinical models of sepsis and endotoxemia.

## Data Availability

All supporting data are included in the article and its additional files.

## Abbreviations

MSCs: Mesenchymal stem cells
SYRCLE: Systematic Review Centre for Laboratory Animal Experimentation
