# Supplementary material for: Efficacy of mesenchymal stem cell therapy for sepsis: a meta-analysis of preclinical studies
Source: Stem Cell Res Ther. 2020 Jun 3;11:214. doi: 10.1186/s13287-020-01730-7 (PMC7268531; doi:10.1186/s13287-020-01730-7)
Supplement: Supplementary file 1 — Additional file 1: Table S1. The detailed search strategy. [file 13287_2020_1730_MOESM1_ESM.doc]

**Additional file 1: Table S1. The detailed search strategy**

| **Electronic databases** | **Search** | **Search strategy** | **Results** |
| --- | --- | --- | --- |
| **Medline** | #1 | "Sepsis" | 74985 |
|  | #2 | (Severe Sepsis) OR Sepsis, Severe) OR Pyemia) OR Pyemias) OR Pyohemia) OR Pyohemias) OR Pyaemia) OR Pyaemias) OR Septicemia) OR Septicemias) OR Poisoning, Blood) OR Blood Poisoning) OR Poisonings, Blood) OR Blood Poisonings) | 89400 |
|  | #3 | #1 OR #2 | 89400 |
|  | #4 | "Mesenchymal Stem Cells" | 52357 |
|  | #5 | (Stem Cell, Mesenchymal) OR Stem Cells, Mesenchymal) OR Mesenchymal Stem Cell) OR Wharton Jelly Cells) OR Wharton's Jelly Cells) OR Wharton's Jelly Cell) OR Whartons Jelly Cells) OR Mesenchymal Stromal Cells) OR Mesenchymal Stromal Cell) OR Stromal Cell, Mesenchymal) OR Stromal Cells, Mesenchymal) OR Adipose-Derived Mesenchymal Stem Cells) OR Adipose Derived Mesenchymal Stem Cells) OR Adipose Tissue-Derived Mesenchymal Stem Cells) OR Adipose Tissue Derived Mesenchymal Stem Cells) OR Adipose-Derived Mesenchymal Stromal Cells) OR Adipose Derived Mesenchymal Stromal Cells) OR Adipose Tissue-Derived Mesenchymal Stromal Cells) OR Adipose Tissue Derived Mesenchymal Stromal Cells) OR Mesenchymal Stem Cells, Adipose-Derived) OR Mesenchymal Stem Cells, Adipose Derived) OR Bone Marrow Stromal Cells, Multipotent) OR Multipotent Bone Marrow Stromal Cells) OR Bone Marrow Stromal Stem Cells) OR Multipotent Mesenchymal Stromal Cells) OR Mesenchymal Stromal Cells, Multipotent) OR Mesenchymal Progenitor Cell) OR Mesenchymal Progenitor Cells) OR Progenitor Cell, Mesenchymal) OR Progenitor Cells, Mesenchymal) OR Bone Marrow Mesenchymal Stem Cells) OR Bone Marrow Stromal Cells) OR Bone Marrow Stromal Cell) | 56964 |
|  | #6 | #4 OR #5 | 56970 |
|  | #7 | #3 AND #6 | **226** |
| **EMBASE** | #1 | 'mesenchymal stem cell'/exp | 55525 |
|  | #2 | 'mesenchymal stem cell': ab, ti | 11728 |
|  | #3 | #1 OR #2 | 58744 |
|  | #4 | 'sepsis'/exp | 260083 |
|  | #5 | sepsis: ab,ti | 142178 |
|  | #6 | #4 OR #5 | 292547 |
|  | #7 | #3 AND #6 | **363** |
| **Cochrane Library** | #1 | (sepsis): ti, ab, kw | 10792 |
|  | #2 | MeSH descriptor: [sepsis] explode all trees | 4179 |
|  | #3 | #1 or #2 | 12682 |
|  | #4 | (Mesenchymal Stem Cells): ti, ab, kw | 1385 |
|  | #5 | MeSH descriptor: [Mesenchymal Stem Cells] explode all trees | 99 |
|  | #6 | #4 or #5 | 1385 |
|  | #7 | #3 and #6 | **13** |
| **Web of Science** | #1 | TOPIC: ("Mesenchymal Stem Cells") | 112,606 |
|  | #2 | TOPIC: ("sepsis") | 178,112 |
|  | #3 | #1 AND #2 | **437** |
