# Supplementary figures and images for: Efficacy of mesenchymal stem cell therapy for sepsis: a meta-analysis of preclinical studies
Source: Stem Cell Res Ther. 2020 Jun 3;11:214. doi: 10.1186/s13287-020-01730-7 (PMC7268531; doi:10.1186/s13287-020-01730-7)

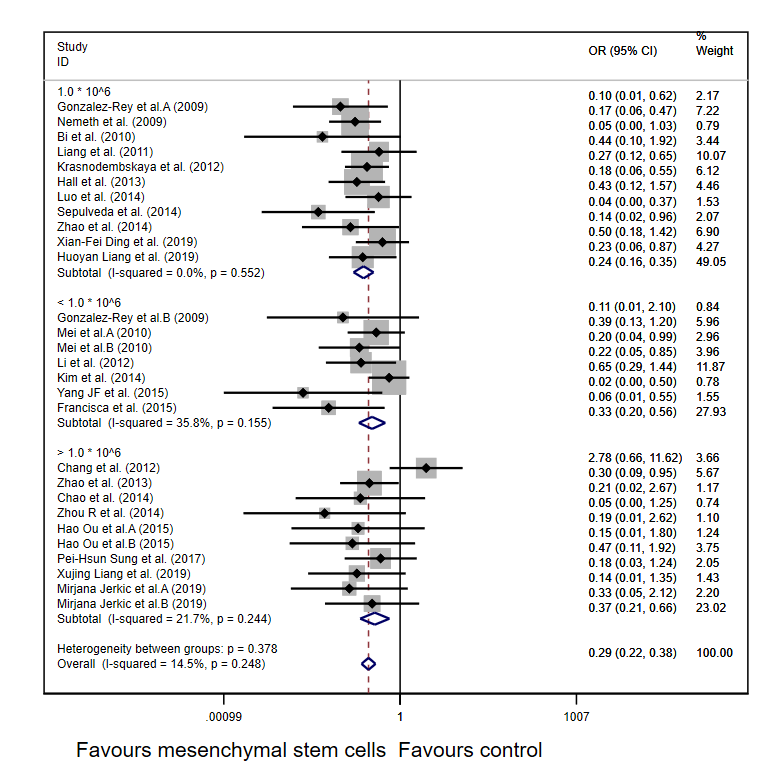

Supplement: Supplementary file 2 — Additional file 2: Fig. S1. Forest plot summarizing the relationship between mesenchymal stem cell dose and mortality in preclinical models of sepsis and endotoxemia. [file 13287_2020_1730_MOESM2_ESM.tif]

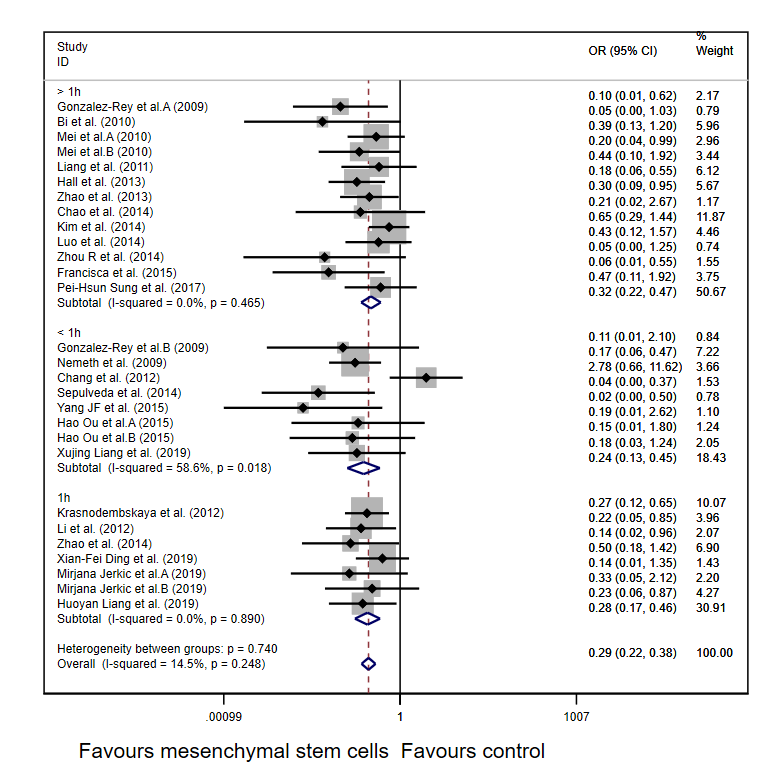

Supplement: Supplementary file 3 — Additional file 3: Fig. S2. Forest plot summarizing the relationship between mesenchymal cell therapy timing of administration and mortality in preclinical models of sepsis and endotoxemia. [file 13287_2020_1730_MOESM3_ESM.tif]

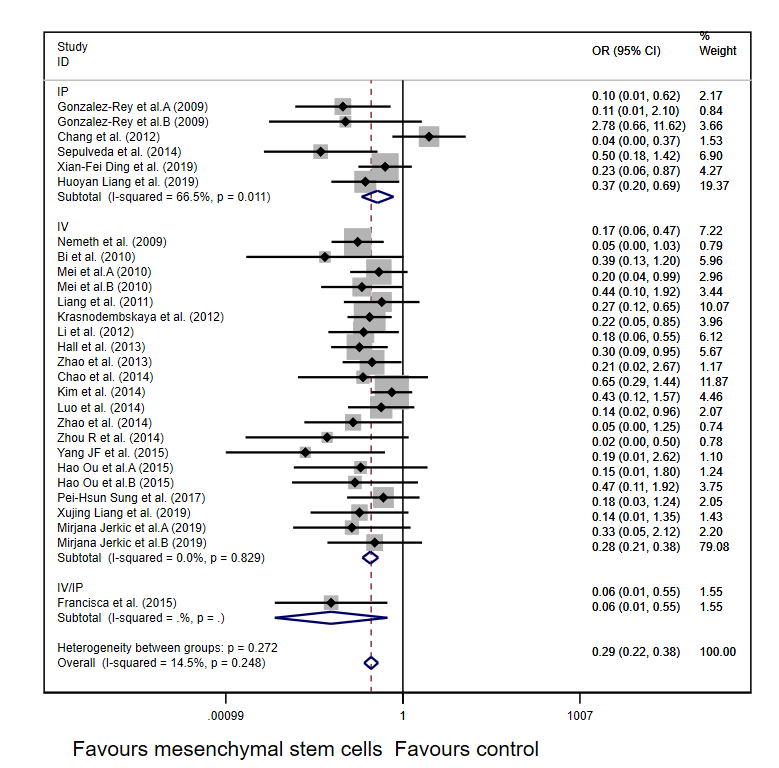

Supplement: Supplementary file 4 — Additional file 4: Fig. S3. Forest plot summarizing the relationship between mesenchymal stem cell administration route (intravenous versus intraperitoneal injection) and mortality in preclinical models of sepsis and endotoxemia. [file 13287_2020_1730_MOESM4_ESM.tif]

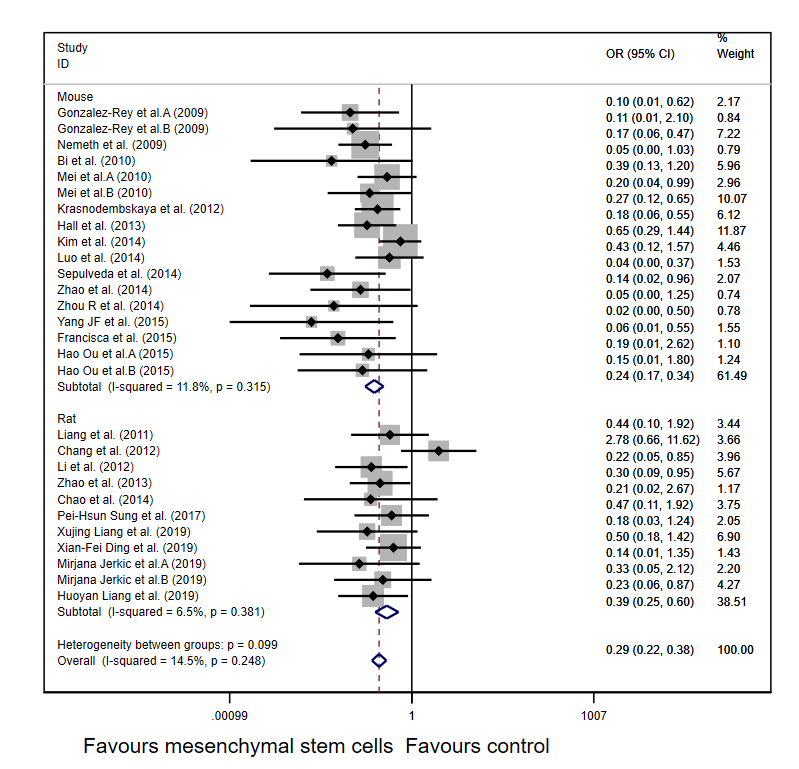

Supplement: Supplementary file 5 — Additional file 5: Fig. S4. Forest plot summarizing the relationship between mesenchymal stem cell-treated animal model species (rat versus mouse) and mortality in preclinical models of sepsis and endotoxemia. [file 13287_2020_1730_MOESM5_ESM.tif]

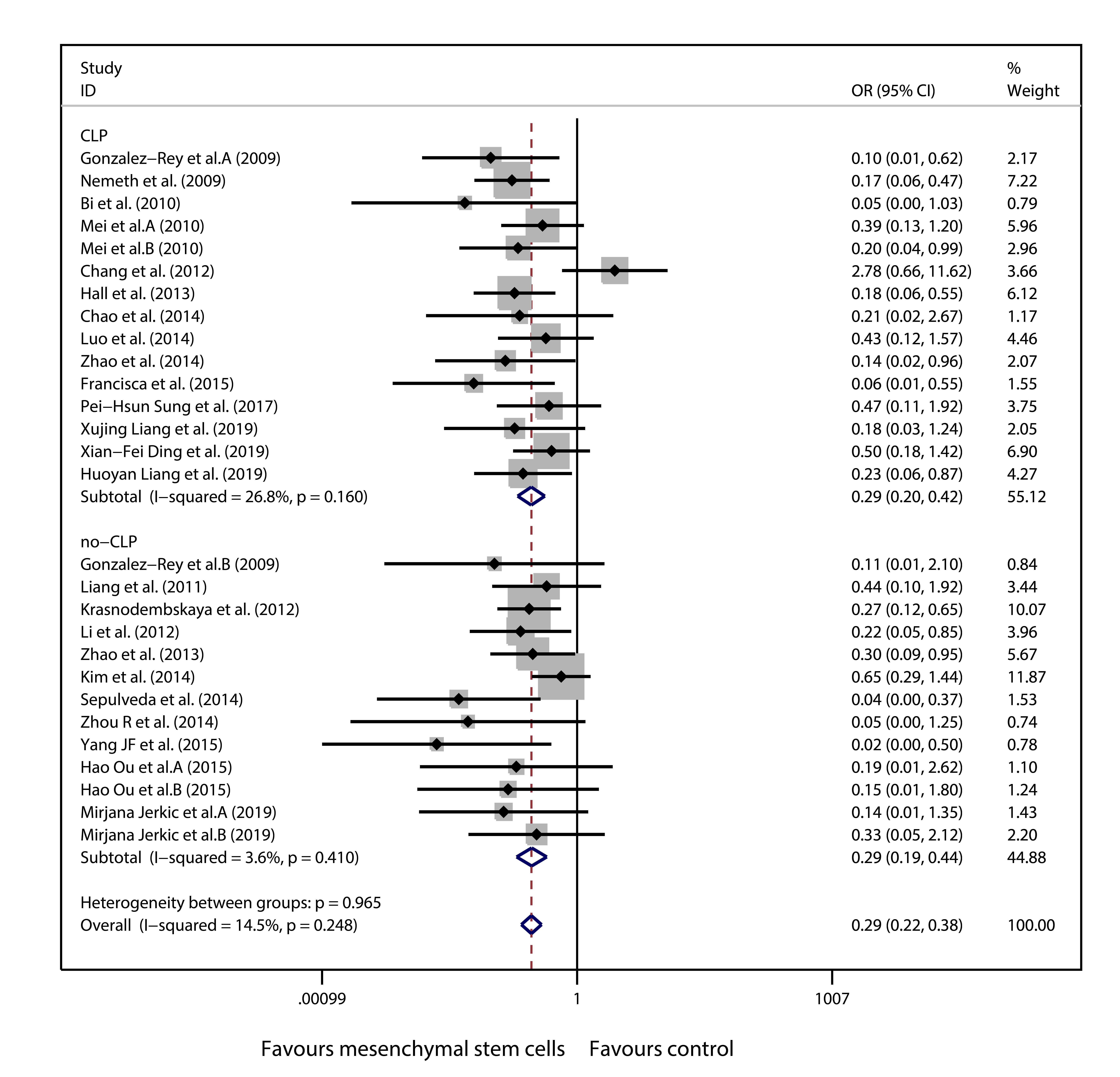

Supplement: Supplementary file 6 — Additional file 6: Fig. S5. Forest plot summarizing the relationship between preclinical models of sepsis and endotoxemia (i.e. cecal ligation and puncture versus live bacteria or bacterial product administration) and mortality following treatment with mesenchymal stem cells. [file 13287_2020_1730_MOESM6_ESM.tif]

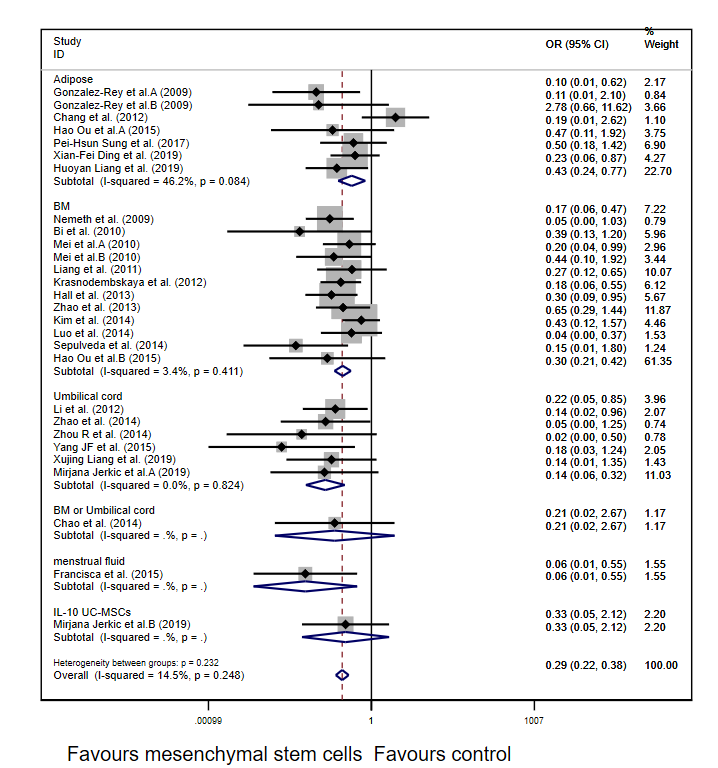

Supplement: Supplementary file 7 — Additional file 7: Fig. S6. Forest plot summarizing the relationship between mesenchymal stem cell source and mortality in preclinical models of sepsis and endotoxemia. [file 13287_2020_1730_MOESM7_ESM.tif]

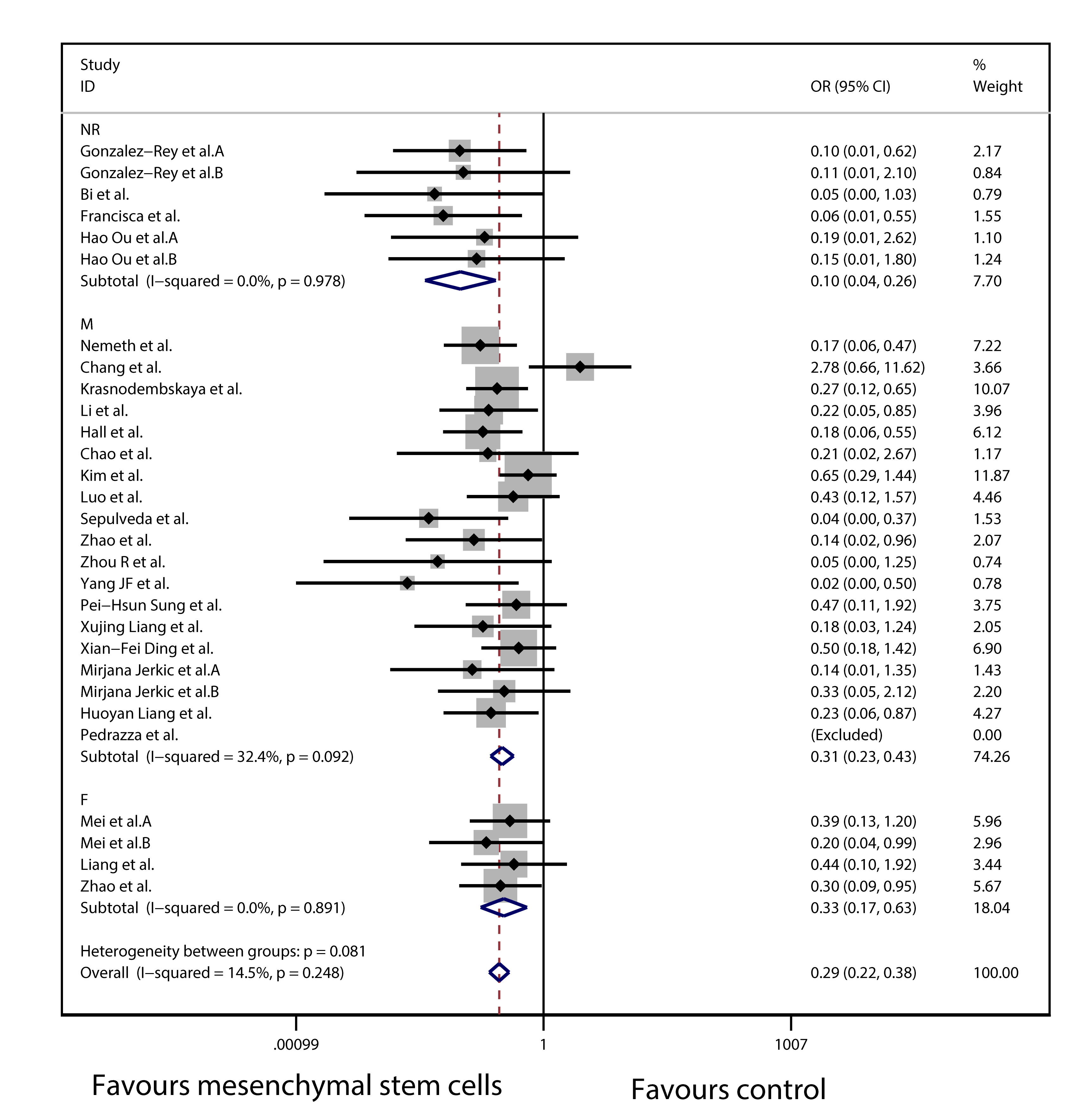

Supplement: Supplementary file 8 — Additional file 8: Fig. S7. Forest plot summarizing the relationship between animal sex and mortality in preclinical models of sepsis and endotoxemia. [file 13287_2020_1730_MOESM8_ESM.tif]
